# Genetic predisposition to smoking in relation to 14 cardiovascular diseases

**DOI:** 10.1093/eurheartj/ehaa193

**Published:** 2020-04-16

**Authors:** Susanna C Larsson, Amy M Mason, Magnus Bäck, Derek Klarin, Scott M Damrauer, Million Veteran Program, Karl Michaëlsson, Stephen Burgess

**Affiliations:** Department of Surgical Sciences, Uppsala University, Uppsala 75185, Sweden; Unit of Cardiovascular and Nutritional Epidemiology, Institute of Environmental Medicine, Karolinska Institutet, Stockholm 17177, Sweden; Department of Public Health and Primary Care, University of Cambridge, Cambridge CB1 8RN, UK; Department of Medicine, Center for Molecular Medicine, Karolinska Institutet, Stockholm 17177, Sweden; Division of Valvular and Coronary Disease, Heart and Vascular Theme, Karolinska University Hospital, Stockholm 14186, Sweden; Boston VA Healthcare System, Boston, MA 02132-4927, USA; Center for Genomic Medicine, Massachusetts General Hospital, Harvard Medical School, Boston, MA 02114, USA; Program in Medical and Population Genetics, Broad Institute of MIT and Harvard, Cambridge, MA 02142, USA; Division of Vascular Surgery and Endovascular Therapy, University of Florida College of Medicine, Gainesville, FL 32610, USA; Corporal Michael J. Crescenz VA Medical Center, Philadelphia, PA 19104, USA; Department of Surgery, Perlman School of Medicine, University of Pennsylvania, Philadelphia, PA 19104, USA; Department of Surgical Sciences, Uppsala University, Uppsala 75185, Sweden; Department of Public Health and Primary Care, University of Cambridge, Cambridge CB1 8RN, UK; MRC Biostatistics Unit, University of Cambridge, Cambridge CB20SR, UK

**Keywords:** Cardiovascular disease, Lifestyle, Mendelian randomization, Risk factors, Single-nucleotide polymorphisms, Smoking

## Abstract

**Aims:**

The aim of this study was to use Mendelian randomization (MR) to determine the causality of the association between smoking and 14 different cardiovascular diseases (CVDs).

**Methods and results:**

Our primary genetic instrument comprised 361 single-nucleotide polymorphisms (SNPs) associated with smoking initiation (ever smoked regularly) at genome-wide significance. Data on the associations between the SNPs and 14 CVDs were obtained from the UK Biobank study (*N* = 367 643 individuals), CARDIoGRAMplusC4D consortium (*N* = 184 305 individuals), Atrial Fibrillation Consortium (2017 dataset; *N* = 154 432 individuals), and Million Veteran Program (MVP; *N* = 190 266 individuals). The main analyses were conducted using the random-effects inverse-variance weighted method and complemented with multivariable MR analyses and the weighted median and MR-Egger approaches. Genetic predisposition to smoking initiation was most strongly and consistently associated with higher odds of coronary artery disease, heart failure, abdominal aortic aneurysm, ischaemic stroke, transient ischaemic attack, peripheral arterial disease, and arterial hypertension. Genetic predisposition to smoking initiation was additionally associated with higher odds of deep vein thrombosis and pulmonary embolism in the UK Biobank but not with venous thromboembolism in the MVP. There was limited evidence of causal associations of smoking initiation with atrial fibrillation, aortic valve stenosis, thoracic aortic aneurysm, and intracerebral and subarachnoid haemorrhage.

**Conclusion:**

This MR study supports a causal association between smoking and a broad range of CVDs, in particular, coronary artery disease, heart failure, abdominal aortic aneurysm, ischaemic stroke, transient ischaemic attack, peripheral arterial disease, and arterial hypertension.

**Figure:**
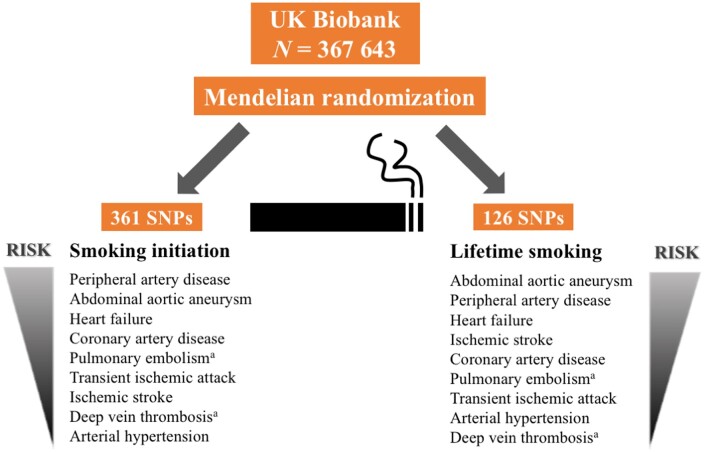

**See page 3311 for the editorial comment on this article (doi: 10.1093/eurheartj/ehaa285)**

## Introduction

A large body of evidence from prospective observational studies indicates that smoking is a risk factor for several cardiovascular diseases (CVDs),^1–6^ but the role of smoking for certain CVDs, such as aortic valve stenosis,^7–9^ atrial fibrillation,^10^ thoracic aortic aneurysm,^11^ ^,^ ^12^ and haemorrhagic stroke^3^ are limited or inconsistent. In contrast to prospective cohort studies,^1^ short-term and long-term follow-up of randomized smoking cessation studies have not revealed a significant effect on fatal coronary heart disease events.^13^ ^,^ ^14^ Given that much of available data on smoking and CVD derive from observational studies, which are unable to fully account for confounding and reverse causality, and the lack of significant effect of smoking cessation on cardiovascular events in interventional trials, the causal nature of the association between smoking and different CVDs remains to be established.

Mendelian randomization (MR) is an analytical method that uses genetic variants, generally single-nucleotide polymorphisms (SNPs), as unbiased proxy indicators for the modifiable risk factor to determine whether the risk factor is a cause of the disease.^15^ ^,^ ^16^ Given that allelic variants are randomly allocated and fixed at conception, MR studies evade reverse causality and are less susceptible to confounding compared with conventional observational studies. To the best of our knowledge, the causal association between smoking and a broad range of CVDs has not been established using MR. We, therefore, applied the MR design to determine the association between smoking and 14 CVD outcomes.

## Methods

### Study design

**Figure 1:**
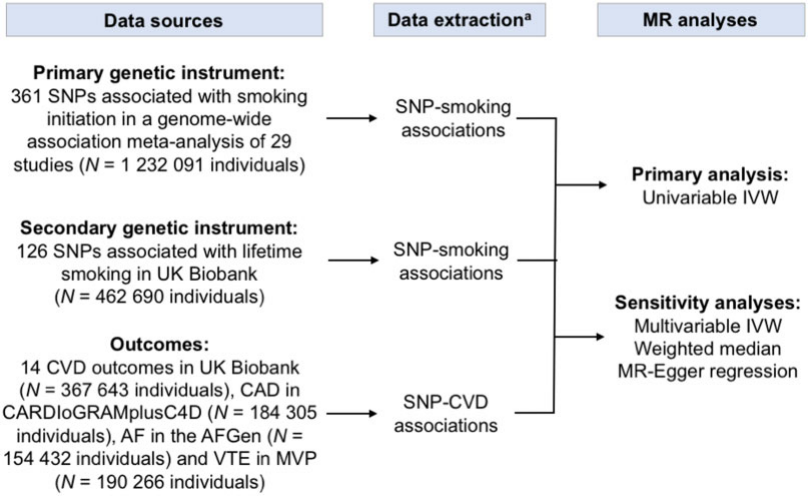
Summary of data sources and methods used in this study. AF, atrial fibrillation; AFGen, Atrial Fibrillation Consortium; CAD, coronary artery disease; CVD, cardiovascular disease; IVW, inverse-variance weighted; MR, Mendelian randomization; MVP, Million Veteran Program; SNP, single-nucleotide polymorphism; VTE, venous thromboembolism. ^a^Data extracted were beta coefficients with corresponding standard errors of the SNP–smoking and SNP–CVD associations.

Summary-level data (i.e. beta coefficients and standard errors) of the associations between SNPs strongly associated with smoking initiation (primary exposure) and lifetime smoking (secondary exposure) were extracted from the hitherto largest genome-wide association studies for these phenotypes (*Figure 1*). The corresponding data for the associations of the smoking-associated SNPs with 14 CVDs were available from UK Biobank (*Figure 1*). In complementary analyses, we obtained summary-level data from genetic consortia of coronary artery disease^17^ atrial fibrillation,^18^ and venous thromboembolism.^19^ Details of the selection of instrumental variables and data sources are provided below.

### Genetic instruments

Instrumental variables for smoking initiation were acquired from a genome-wide association meta-analysis of 29 studies, comprising a total of 1 232 091 European-descent individuals of which 383 631 individuals were part of the UK Biobank. That meta-analysis identified 378 conditionally independent genome-wide significant SNPs at 259 loci (defined as a 1MB region nearby the top *P*-value) associated with smoking initiation, a binary phenotype representing whether an individual had ever smoked cigarettes regularly in their life (current or former).^20^ Information about pipes/cigar/chew or other non-cigarette forms of tobacco use was not included.^20^ The smoking-associated SNPs explained 2.3% of the phenotypic variation.^20^ All but one of the SNPs were available in the UK Biobank. Linkage disequilibrium (defined as *r* ^2^ > 0.1 in European populations) between SNPs was evaluated using LDlink^21^ and was detected among 16 SNP pairs. The SNP with the largest *P*-value was omitted, leaving 361 SNPs as instrumental variables for smoking initiation. Genetic associations with smoking were reported in standard deviation units.^20^ Hence, the reported odds ratios correspond to the increase of 1 SD in prevalence of smoking initiation.

In a supplementary analysis, a genetic instrument for lifetime smoking exposure that takes into account smoking status as well as smoking duration, heaviness, and cessation in ever smokers was applied.^22^ This genetic instrument consists of 126 genome-wide significant SNPs associated with lifetime smoking exposure in UK Biobank (*N* = 462 690).^22^

### Outcome data

Summary-level data for the smoking-associated SNPs with the 14 CVDs came from the UK Biobank, a cohort study of about 500 000 adults, aged 37–73 years and enrolled between 2006 and 2010.^23^ We excluded non-White European participants (to minimize confounding by ancestry), those with relatedness of third degree or higher, low genotype call rate (three or more standard deviations from the mean) and excess heterozygosity, resulting in a final study sample of 367 643 individuals. In this study, participants were followed up until 31 March 2017 or the date of death (recorded until 14 February 2018). The median follow-up was 8.0 years. Outcomes were defined based on electronic health records, hospital procedure codes, and self-reported information validated by interview with a nurse (Supplementary material online, *Table S1*). Logistic regression with adjustment for ten genetic principal components was applied to obtain the beta coefficients and standard errors for the SNP–CVD associations. The SNPs exploited as instrumental variables and their associations with each CVD outcome are presented in Supplementary material online, *Table S2*.

Summary-level data for coronary artery disease, atrial fibrillation, and venous thromboembolism were additionally obtained from the Coronary ARtery DIsease Genome-wide Replication and Meta-analysis plus The Coronary Artery Disease Genetics (CARDIoGRAMplusC4D) consortium (60 801 cases and 123 504 non-cases),^17^ the Atrial Fibrillation Consortium (22 346 cases and 132 086 non-cases; AFGen exome chip analysis, not including UK Biobank),^18^ and the Million Veteran Program (MVP) (8929 cases and 181 337 non-cases), respectively.^19^ There was no participant overlap between those three datasets and the UK Biobank. For comparison, we also present previously reported results for ischaemic stroke based on the MEGASTROKE consortium (34 217 ischaemic stroke cases and 404 630 non-cases, not including UK Biobank).^24^ ^,^ ^25^

The UK Biobank study was approved by the North West Multicenter Research Ethics Committee. Original studies included in the consortia had been approved by a relevant review board. The present analyses were approved by the Swedish Ethical Review Authority.

### Statistical analysis

The inverse-variance-weighted (IVW) method, under a multiplicative random-effects model, was used to obtain the primary causal estimates. Ratio estimates are calculated for each SNP as the beta coefficient for the SNP–CVD association divided by the beta coefficient for the SNP–smoking association. These estimates are then combined across SNPs in a random-effects IVW meta-analysis. This MR method provides the highest precision but assumes that all SNPs are valid instrumental variables. Heterogeneity among estimates based on individual SNPs was assessed with the *I* ^2^ statistic.^26^

Multivariable MR analyses^27^ were performed to adjust for potential confounders that are genetically correlated with smoking initiation, including alcohol drinking (*r*_g_ = 0.36), body mass index (*r*_g_ = 0.12), and years of education (*r*_g_ = −0.40).^20^ Multivariable MR analysis was also used to assess whether the associations between genetic predisposition to smoking initiation and CVD outcomes that are comorbid or secondary to coronary artery disease^28^ may be driven by coronary artery disease.

The weighted median and MR-Egger regression methods were used as sensitivity analyses. The weighted median approach gives consistent estimates if at least 50% of the weight in the analysis comes from valid instrumental variables.^29^ The MR-Egger approach can detect and correct for directional pleiotropy but suffers from low power.^29^ In a further sensitivity analysis, the associations between genetic predisposition to smoking initiation and CVD outcomes were assessed in never smokers (based on self-report) in UK Biobank.

To correct for multiple testing, Bonferroni correction was applied and two-sided *P*-values <3.6× 10^−3^ (where α = 0.05/14 CVD outcomes) were deemed statistically significant and also considered strong evidence of a causal association. Findings with *P*-values between 0.05 and 3.6 × 10^−3^ were regarded as suggestive evidence of association. The analyses were performed using the mrrobust^30^ and MendelianRandomization^31^ packages.

## Results

### Participants

In the analytic sample of 367 643 UK Biobank participants, the mean age was 57 years (5th–95th percentile: 43–69 years) and 46% were men. The prevalence of smoking initiation (ever smokers) was 46%. Around 93% were alcohol drinkers.

### Smoking and cardiovascular disease

**Figure 2:**
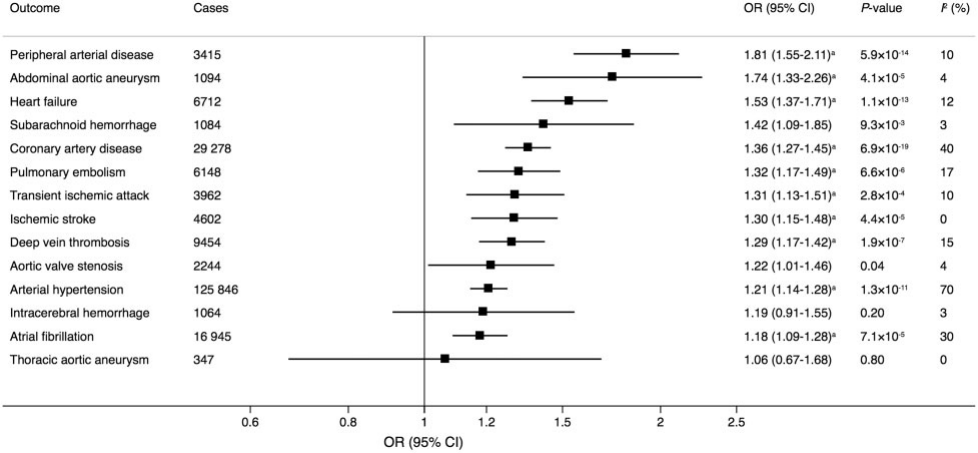
Associations of genetic predisposition to smoking initiation with 14 cardiovascular diseases in UK Biobank. The odds ratios correspond to the increase of one standard deviation in prevalence of smoking initiation (ever smoked regularly). Estimates are from the multiplicative random-effects inverse variance-weighted method. CI, confidence interval; OR, odds ratio. The *I* ^2^ statistic quantifies the amount of heterogeneity among estimates based on individual SNPs. ^a^ Significant at the Bonferroni-corrected threshold of *P *<* *3.6 × 10^−3^.

**Figure 3:**
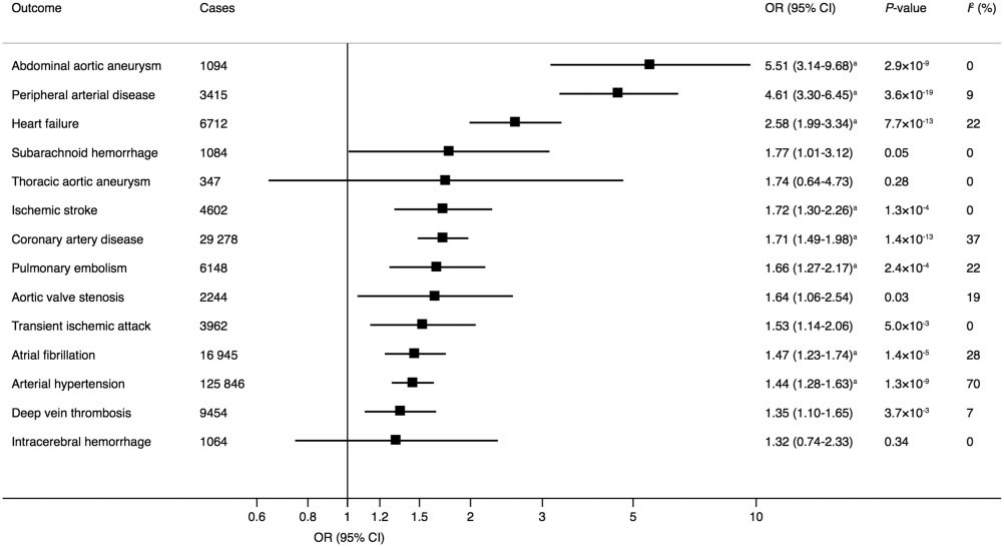
Associations of genetically predicted lifetime smoking index with 14 cardiovascular diseases in UK Biobank. Odds ratios are expressed per one standard deviation increase of the lifetime smoking index. Estimates are from the multiplicative random-effects inverse variance-weighted method. CI, confidence interval; OR, odds ratio. The *I* ^2^ statistic quantifies the amount of heterogeneity among estimates based on individual SNPs individual SNPs. ^a^Significant at the Bonferroni-corrected threshold of *P *<* *3.6 × 10^−3^.

In the univariable IVW analysis, genetic predisposition to smoking initiation was statistically significantly positively associated with 10 of the 14 outcomes in UK Biobank (*Figure 2*). The odds ratios ranged from 1.18 (95% confidence interval 1.09–1.28; *P *=* *7.1 × 10^−5^) for atrial fibrillation to 1.81 (95% confidence interval 1.55–2.11; *P *=* *5.2 × 10^−7^) for peripheral arterial disease. In the multivariable IVW models adjusting for genetically correlated phenotypes (i.e. alcohol consumption, body mass index, and education), the associations remained statistically significant for coronary artery disease, heart failure, abdominal aortic aneurysm, deep vein thrombosis, pulmonary embolism, peripheral arterial disease, and arterial hypertension; suggestive evidence of associations remained for ischaemic stroke, transient ischaemic attack, and atrial fibrillation (Supplementary material online, *Table S3*). The association of genetic predisposition to smoking initiation with atrial fibrillation did not persist after adjustment for coronary artery disease (Supplementary material online, *Table S3*). The results were similar in the weighted median analysis and no evidence of directional pleiotropy was detected in the MR-Egger regression analysis (all *P *≥* *0.15) (Supplementary material online, *Table S3*). In the analysis confined to never smokers, all associations except the association with arterial hypertension were null in the univariable or multivariable IVW models (Supplementary material online, *Table S4*). Genetic predisposition to smoking initiation was also positively associated with coronary artery disease and ischaemic stroke in the CARDIoGRAMplusC4D consortium and MEGASTROKE,^25^ respectively, but the associations were weaker than in UK Biobank (Supplementary material online, *Table S3*). There were no robust associations of genetic predisposition to smoking initiation with atrial fibrillation or venous thromboembolism in the AFGen and MVP, respectively (Supplementary material online, *Table S3*). Findings for the lifetime smoking exposure in UK Biobank (*Figure 3*) and the consortia and MVP (Supplementary material online, *Table S5*) were broadly similar to those for smoking initiation.

For the observed smoking initiation–CVD associations in UK Biobank (*Take home figure*), we estimated the predicted risk reduction that would result from a reduction in the prevalence of smoking from 46% (the observed prevalence in UK Biobank) to 20%. This corresponds to a 1.22 unit change in the log-odds of smoking, a difference similar to the difference in log odds between those in the 1st and 99th percentiles of the genetic risk score for smoking initiation. This risk reduction was 52% for peripheral arterial disease, 49% for abdominal aortic aneurysm, 41% for heart failure, 32% for coronary artery disease, 29% for pulmonary embolism, 28% for transient ischaemic attack, 27% for ischaemic stroke and deep vein thrombosis, and 21% for arterial hypertension.

**Take home figure:**
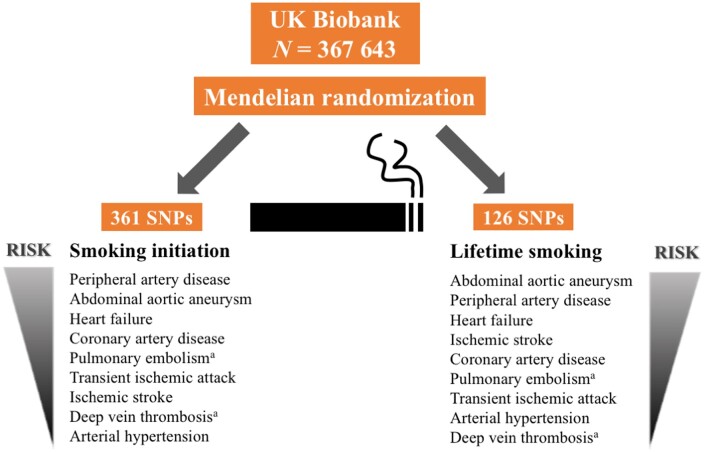
Observed associations of genetic predisposition to smoking initiation and lifetime smoking with cardiovascular diseases in UK Biobank. ^a^No robust association was observed between smoking and venous thromboembolism (pulmonary embolism and deep vein thrombosis combined) in the Million Veteran Program.

## Discussion

### Principal findings

This MR study showed that genetic predisposition to smoking is associated with an increased risk of a broad range of CVDs (*Take home figure*). The strongest and most consistent positive associations were observed between smoking initiation and coronary artery disease, heart failure, abdominal aortic aneurysm, ischaemic stroke, transient ischaemic attack, peripheral arterial disease, and arterial hypertension. Smoking initiation was additionally associated with deep vein thrombosis and pulmonary embolism in the UK Biobank but not with venous thromboembolism in the MVP. There was limited evidence of causal associations of smoking initiation with atrial fibrillation, aortic valve stenosis, thoracic aortic aneurysm, and intracerebral and subarachnoid haemorrhage.

The results from the present MR study based on data from UK Biobank corroborate the findings of conventional prospective observational studies showing that smoking is a risk factor for coronary artery disease,^1^ heart failure,^4^ abdominal aortic aneurysm,^5^ ischaemic stroke,^3^ peripheral arterial disease,^2^ deep vein thrombosis,^6^ and pulmonary embolism^6^ (Supplementary material online, *Figure S1*). However, this and a previous MR study based on another study sample (*n* = 1545 cases of intracerebral haemorrhage and 1481 controls)^25^ found limited evidence of a causal association between smoking and intracerebral haemorrhage and subarachnoid haemorrhage. These findings are in contrast to those of a large prospective study and meta-analysis which showed that smoking was associated with increased risk of both intracerebral haemorrhage and subarachnoid haemorrhage^3^ (Supplementary material online, *Figure S1*). The present and the prior MR study^25^ may have been unable to observe a statistically significant association due to small number of cases of haemorrhagic stroke. With regard to ischaemic stroke subtypes, smoking has been shown to increase the risk of large artery and small vessel stroke but not cardioembolic stroke.^25^ Data on ischaemic stroke subtypes were not available in UK Biobank.

In contrast to abdominal aortic aneurysm, we found no evidence of a causal association between smoking and thoracic aortic aneurysm but owing to the limited number of cases, we cannot rule out that we may have overlooked a weak association. Observational studies of the association between smoking and thoracic aortic aneurysm are scarce. No association between smoking and risk of rupture or dissection of thoracic aortic aneurysm was observed in a cohort of thoracic aortic aneurysm patients without history of aortic dissection.^11^ However, smoking was associated with a 2.2-fold higher risk of thoracic aortic aneurysm in a cohort of 30 447 adults, including 45 thoracic aortic aneurysm cases.^12^ Thus, the role of smoking for thoracic aortic aneurysm requires further study.

Observational studies of smoking in relation to risk of aortic valve stenosis^7–9^ and atrial fibrillation^10^ are limited or inconsistent. One large^9^ and two small prospective cohort studies^7^ ^,^ ^8^ found a 30%^9^ to almost two-fold^7^ increased risk of aortic valve stenosis associated with smoking, and a meta-analysis found an overall 32% increased risk of atrial fibrillation for current vs. never smokers.^10^ This MR study found no association between smoking initiation and aortic valve stenosis after adjustment for body mass index, which is a strong risk factor for aortic valve stenosis.^32^ Likewise, no consistent association was observed between smoking initiation and atrial fibrillation.

### Potential mechanisms

Cigarette smoke can damage the cardiovascular system through its content of oxidant gases (e.g. oxides of nitrogen and free radicals) and other toxic substances, which may increase the risk of CVD through lipid oxidation, endothelial dysfunction, inflammation platelet activation, thrombogenesis, and augmented coagulability.^33^ Furthermore, nicotine may provoke acute cardiovascular events by increasing myocardial contractility and vasoconstriction, leading to increased myocardial work and oxygen demand as well as reduced coronary and cerebral blood flow.^33^ Nicotine exposure may further promote aneurysmal rupture through actions on vascular smooth muscle cell nicotinic acetylcholine receptors containing α7 subunits.^34^ Smoking might also affect CVD risk through antioestrogenic effects, observed in both women and men.^35–37^

### Strength and limitations

This study has several strengths. First, the MR design reduces the possibility that the observed associations were biased by reverse causality and confounding. Second, the genetic instrument for smoking initiation comprised multiple SNPs robustly associated with smoking, thereby providing a strong genetic instrument. Third, the analyses included a large sample size and generally large number of CVD events, and therefore, the statistical power was high in most analyses. Fourth, by restricting the study sample to individuals of European-descent in UK Biobank, population stratification bias was minimized.

This study also has several limitations. First, the possibility that the smoking-related SNPs affect CVD outcomes through other causal pathways than through smoking exposure cannot be entirely ruled out. Nonetheless, in never smokers the associations of genetic predisposition to smoking initiation with the CVD outcomes were generally null (except for arterial hypertension) in the crude or multivariable MR analysis. The weak associations for some CVD outcomes may be due to exposure to cigarette smoke amongst never smokers or to misclassification of smoking status (ever smokers incorrectly reported never smoked regularly) as self-reported smoking prevalence is known to be lower than directly measured values of cotinine levels.^38^

Second, the causal association between smoking cessation and CVD could not be assessed because identified SNPs for smoking cessation are few and explains a small proportion of the phenotypic variance (0.1%).^20^ To address those limitations, we conducted a complementary analysis of lifetime smoking index, which takes into account smoking duration, heaviness, and cessation,^22^ and observed similar results to those based on the smoking initiation phenotype.

A third limitation is that participants of the UK Biobank were included in both the exposure and outcome datasets (about 30% participant overlap) in the primary analyses. This overlap may have introduced some bias in the causal estimates in the direction of the observational association between smoking and CVD risk.^39^ However, the genetic variants are reasonably strongly associated with the exposure (F statistic 78.5), meaning that bias due to sample overlap is reasonably small. The associations of genetic predisposition to smoking initiation with coronary artery disease, ischaemic stroke, atrial fibrillation, and venous thromboembolism were stronger in UK Biobank than in non-overlapping sets of individuals included in the consortia. This might be related to more consistent case definitions in UK Biobank, population mixture in the consortia (as the genetic variants were chosen based on their associations with smoking status in Europeans, we may expect associations to be attenuated in a mixed ethnicity population), or differences in smoking status and prevalence of other risk factors in different populations. For example, the MVP cohort consists of over 90% men and the prevalence of ever smoking (about 75%) and CVD risk factors, such as Type 2 diabetes, hyperlipidaemia, and obesity, is much higher in the MVP cohort than in the UK Biobank.^19^ As environmental influences on smoking are stronger in this cohort, genetic influences on smoking are likely to be weaker.

Finally, the power was insufficient for analyses of the association of genetic predisposition to smoking with CVD in individuals of non-European ancestry. Genome-wide associations studies assessing the genetics of smoking in non-Europeans, as well as large MR studies of smoking and CVD risk in other populations, are warranted.

## Conclusions

This study supports a causal association between smoking and a broad range of CVDs, in particular, coronary artery disease, heart failure, abdominal aortic aneurysm, ischaemic stroke, transient ischaemic attack, peripheral arterial disease, and arterial hypertension. These findings add to the level of evidence that smoking is a causal risk factor for CVD.^40^

## Supplementary Material

ehaa193_Supplementary_Data
