# Supplementary material for: Genetic predisposition to smoking in relation to 14 cardiovascular diseases
Source: Eur Heart J. 2020 Apr 16;41(35):3304–10. doi: 10.1093/eurheartj/ehaa193 (PMC7544540; doi:10.1093/eurheartj/ehaa193)
Supplement: ehaa193_Supplementary_Data [file ehaa193_supplementary_data.zip › ehaa193-suppl_data/Supplement 1.docx]

**Supplementary material online:**

**Genetic predisposition to smoking in relation to 14 cardiovascular diseases**

Susanna C. Larsson, Amy M. Mason, Magnus Bäck, Derek Klarin, Scott M. Damrauer, Million Veteran Program, Karl Michaëlsson, and Stephen Burgess

*Supplementary Figure 1.* Association between smoking and cardiovascular diseases in meta-analyses of prospective studies or individual large prospective studies if no meta-analysis was available. CI, confidence interval; CVD, cardiovascular disease; N=number of studies included in the meta-analysis; NA, not available; Ref, reference; RR, relative risk.

**References**

1. Mons U, Muezzinler A, Gellert C, Schottker B, Abnet CC, Bobak M, de Groot L, Freedman ND, Jansen E, Kee F, Kromhout D, Kuulasmaa K, Laatikainen T, O'Doherty MG, Bueno-de-Mesquita B, Orfanos P, Peters A, van der Schouw YT, Wilsgaard T, Wolk A, Trichopoulou A, Boffetta P, Brenner H, Consortium C. Impact of smoking and smoking cessation on cardiovascular events and mortality among older adults: meta-analysis of individual participant data from prospective cohort studies of the CHANCES consortium. *BMJ* 2015;**350**:h1551.

2. Lu L, Mackay DF, Pell JP. Meta-analysis of the association between cigarette smoking and peripheral arterial disease. *Heart* 2014;**100**:414-423.

3. Price AJ, Wright FL, Green J, Balkwill A, Kan SW, Yang TO, Floud S, Kroll ME, Simpson R, Sudlow CLM, Beral V, Reeves GK. Differences in risk factors for 3 types of stroke: UK prospective study and meta-analyses. *Neurology* 2018;**90**:e298-e306.

4. Aune D, Schlesinger S, Norat T, Riboli E. Tobacco smoking and the risk of heart failure: A systematic review and meta-analysis of prospective studies. *Eur J Prev Cardiol* 2019;**26**:279-288.

5. Aune D, Schlesinger S, Norat T, Riboli E. Tobacco smoking and the risk of abdominal aortic aneurysm: a systematic review and meta-analysis of prospective studies. *Sci Rep* 2018;**8**:14786.

6. Gregson J, Kaptoge S, Bolton T, Pennells L, Willeit P, Burgess S, Bell S, Sweeting M, Rimm EB, Kabrhel C, Zoller B, Assmann G, Gudnason V, Folsom AR, Arndt V, Fletcher A, Norman PE, Nordestgaard BG, Kitamura A, Mahmoodi BK, Whincup PH, Knuiman M, Salomaa V, Meisinger C, Koenig W, Kavousi M, Volzke H, Cooper JA, Ninomiya T, Casiglia E, Rodriguez B, Ben-Shlomo Y, Despres JP, Simons L, Barrett-Connor E, Bjorkelund C, Notdurfter M, Kromhout D, Price J, Sutherland SE, Sundstrom J, Kauhanen J, Gallacher J, Beulens JWJ, Dankner R, Cooper C, Giampaoli S, Deen JF, Gomez de la Camara A, Kuller LH, Rosengren A, Svensson PJ, Nagel D, Crespo CJ, Brenner H, Albertorio-Diaz JR, Atkins R, Brunner EJ, Shipley M, Njolstad I, Lawlor DA, van der Schouw YT, Selmer RM, Trevisan M, Verschuren WMM, Greenland P, Wassertheil-Smoller S, Lowe GDO, Wood AM, Butterworth AS, Thompson SG, Danesh J, Di Angelantonio E, Meade T, Emerging Risk Factors C. Cardiovascular Risk Factors Associated With Venous Thromboembolism. *JAMA Cardiol* 2019;**4**:163-173.

7. Martinsson A, Ostling G, Persson M, Sundquist K, Andersson C, Melander O, Engstrom G, Hedblad B, Smith JG. Carotid plaque, intima-media thickness, and incident aortic stenosis: a prospective cohort study. *Arterioscler Thromb Vasc Biol* 2014;**34**:2343-2348.

8. Eveborn GW, Schirmer H, Lunde P, Heggelund G, Hansen JB, Rasmussen K. Assessment of risk factors for developing incident aortic stenosis: the Tromso Study. *Eur J Epidemiol* 2014;**29**:567-575.

9. Larsson SC, Wolk A, Back M. Alcohol consumption, cigarette smoking and incidence of aortic valve stenosis. *J Intern Med* 2017;**282**:332-339.

10. Aune D, Schlesinger S, Norat T, Riboli E. Tobacco smoking and the risk of atrial fibrillation: A systematic review and meta-analysis of prospective studies. *Eur J Prev Cardiol* 2018;**25**:1437-1451.

11. Kim JB, Kim K, Lindsay ME, MacGillivray T, Isselbacher EM, Cambria RP, Sundt TM, 3rd. Risk of rupture or dissection in descending thoracic aortic aneurysm. *Circulation* 2015;**132**:1620-1629.

12. Landenhed M, Engstrom G, Gottsater A, Caulfield MP, Hedblad B, Newton-Cheh C, Melander O, Smith JG. Risk profiles for aortic dissection and ruptured or surgically treated aneurysms: a prospective cohort study. *J Am Heart Assoc* 2015;**4**:e001513.

13. Rose G, Colwell L. Randomised controlled trial of anti-smoking advice: final (20 year) results. *J Epidemiol Community Health* 1992;**46**:75-77.

14. Multiple risk factor intervention trial. Risk factor changes and mortality results. Multiple Risk Factor Intervention Trial Research Group. *JAMA* 1982;**248**:1465-1477.

15. Davey Smith G, Ebrahim S. 'Mendelian randomization': can genetic epidemiology contribute to understanding environmental determinants of disease? *Int J Epidemiol* 2003;**32**:1-22.

16. Burgess S, Thompson SG. *Mendelian randomization: methods for using genetic variants in causal estimation.* Chapman and Hall/CRC Press 2015.

17. Nikpay M, Goel A, Won HH, Hall LM, Willenborg C, Kanoni S, Saleheen D, Kyriakou T, Nelson CP, Hopewell JC, Webb TR, Zeng L, Dehghan A, Alver M, Armasu SM, Auro K, Bjonnes A, Chasman DI, Chen S, Ford I, Franceschini N, Gieger C, Grace C, Gustafsson S, Huang J, Hwang SJ, Kim YK, Kleber ME, Lau KW, Lu X, Lu Y, Lyytikainen LP, Mihailov E, Morrison AC, Pervjakova N, Qu L, Rose LM, Salfati E, Saxena R, Scholz M, Smith AV, Tikkanen E, Uitterlinden A, Yang X, Zhang W, Zhao W, de Andrade M, de Vries PS, van Zuydam NR, Anand SS, Bertram L, Beutner F, Dedoussis G, Frossard P, Gauguier D, Goodall AH, Gottesman O, Haber M, Han BG, Huang J, Jalilzadeh S, Kessler T, Konig IR, Lannfelt L, Lieb W, Lind L, Lindgren CM, Lokki ML, Magnusson PK, Mallick NH, Mehra N, Meitinger T, Memon FU, Morris AP, Nieminen MS, Pedersen NL, Peters A, Rallidis LS, Rasheed A, Samuel M, Shah SH, Sinisalo J, Stirrups KE, Trompet S, Wang L, Zaman KS, Ardissino D, Boerwinkle E, Borecki IB, Bottinger EP, Buring JE, Chambers JC, Collins R, Cupples LA, Danesh J, Demuth I, Elosua R, Epstein SE, Esko T, Feitosa MF, Franco OH, Franzosi MG, Granger CB, Gu D, Gudnason V, Hall AS, Hamsten A, Harris TB, Hazen SL, Hengstenberg C, Hofman A, Ingelsson E, Iribarren C, Jukema JW, Karhunen PJ, Kim BJ, Kooner JS, Kullo IJ, Lehtimaki T, Loos RJ, Melander O, Metspalu A, Marz W, Palmer CN, Perola M, Quertermous T, Rader DJ, Ridker PM, Ripatti S, Roberts R, Salomaa V, Sanghera DK, Schwartz SM, Seedorf U, Stewart AF, Stott DJ, Thiery J, Zalloua PA, O'Donnell CJ, Reilly MP, Assimes TL, Thompson JR, Erdmann J, Clarke R, Watkins H, Kathiresan S, McPherson R, Deloukas P, Schunkert H, Samani NJ, Farrall M, Consortium CAD. A comprehensive 1,000 Genomes-based genome-wide association meta-analysis of coronary artery disease. *Nat Genet* 2015;**47**:1121-1130.

18. Christophersen IE, Rienstra M, Roselli C, Yin X, Geelhoed B, Barnard J, Lin H, Arking DE, Smith AV, Albert CM, Chaffin M, Tucker NR, Li M, Klarin D, Bihlmeyer NA, Low SK, Weeke PE, Muller-Nurasyid M, Smith JG, Brody JA, Niemeijer MN, Dorr M, Trompet S, Huffman J, Gustafsson S, Schurmann C, Kleber ME, Lyytikainen LP, Seppala I, Malik R, Horimoto A, Perez M, Sinisalo J, Aeschbacher S, Theriault S, Yao J, Radmanesh F, Weiss S, Teumer A, Choi SH, Weng LC, Clauss S, Deo R, Rader DJ, Shah SH, Sun A, Hopewell JC, Debette S, Chauhan G, Yang Q, Worrall BB, Pare G, Kamatani Y, Hagemeijer YP, Verweij N, Siland JE, Kubo M, Smith JD, Van Wagoner DR, Bis JC, Perz S, Psaty BM, Ridker PM, Magnani JW, Harris TB, Launer LJ, Shoemaker MB, Padmanabhan S, Haessler J, Bartz TM, Waldenberger M, Lichtner P, Arendt M, Krieger JE, Kahonen M, Risch L, Mansur AJ, Peters A, Smith BH, Lind L, Scott SA, Lu Y, Bottinger EB, Hernesniemi J, Lindgren CM, Wong JA, Huang J, Eskola M, Morris AP, Ford I, Reiner AP, Delgado G, Chen LY, Chen YI, Sandhu RK, Li M, Boerwinkle E, Eisele L, Lannfelt L, Rost N, Anderson CD, Taylor KD, Campbell A, Magnusson PK, Porteous D, Hocking LJ, Vlachopoulou E, Pedersen NL, Nikus K, Orho-Melander M, Hamsten A, Heeringa J, Denny JC, Kriebel J, Darbar D, Newton-Cheh C, Shaffer C, Macfarlane PW, Heilmann-Heimbach S, Almgren P, Huang PL, Sotoodehnia N, Soliman EZ, Uitterlinden AG, Hofman A, Franco OH, Volker U, Jockel KH, Sinner MF, Lin HJ, Guo X, Dichgans M, Ingelsson E, Kooperberg C, Melander O, Loos RJF, Laurikka J, Conen D, Rosand J, van der Harst P, Lokki ML, Kathiresan S, Pereira A, Jukema JW, Hayward C, Rotter JI, Marz W, Lehtimaki T, Stricker BH, Chung MK, Felix SB, Gudnason V, Alonso A, Roden DM, Kaab S, Chasman DI, Heckbert SR, Benjamin EJ, Tanaka T, Lunetta KL, Lubitz SA, Ellinor PT. Large-scale analyses of common and rare variants identify 12 new loci associated with atrial fibrillation. *Nat Genet* 2017;**49**:946-952.

19. Klarin D, Busenkell E, Judy R, Lynch J, Levin M, Haessler J, Aragam K, Chaffin M, Haas M, Lindstrom S, Assimes TL, Huang J, Min Lee K, Shao Q, Huffman JE, Kabrhel C, Huang Y, Sun YV, Vujkovic M, Saleheen D, Miller DR, Reaven P, DuVall S, Boden WE, Pyarajan S, Reiner AP, Tregouet DA, Henke P, Kooperberg C, Gaziano JM, Concato J, Rader DJ, Cho K, Chang KM, Wilson PWF, Smith NL, O'Donnell CJ, Tsao PS, Kathiresan S, Obi A, Damrauer SM, Natarajan P, Consortium I, Veterans Affairs' Million Veteran P. Genome-wide association analysis of venous thromboembolism identifies new risk loci and genetic overlap with arterial vascular disease. *Nat Genet* 2019;**51**:1574-1579.

20. Liu M, Jiang Y, Wedow R, Li Y, Brazel DM, Chen F, Datta G, Davila-Velderrain J, McGuire D, Tian C, Zhan X, andMe Research T, Psychiatry HA-I, Choquet H, Docherty AR, Faul JD, Foerster JR, Fritsche LG, Gabrielsen ME, Gordon SD, Haessler J, Hottenga JJ, Huang H, Jang SK, Jansen PR, Ling Y, Magi R, Matoba N, McMahon G, Mulas A, Orru V, Palviainen T, Pandit A, Reginsson GW, Skogholt AH, Smith JA, Taylor AE, Turman C, Willemsen G, Young H, Young KA, Zajac GJM, Zhao W, Zhou W, Bjornsdottir G, Boardman JD, Boehnke M, Boomsma DI, Chen C, Cucca F, Davies GE, Eaton CB, Ehringer MA, Esko T, Fiorillo E, Gillespie NA, Gudbjartsson DF, Haller T, Harris KM, Heath AC, Hewitt JK, Hickie IB, Hokanson JE, Hopfer CJ, Hunter DJ, Iacono WG, Johnson EO, Kamatani Y, Kardia SLR, Keller MC, Kellis M, Kooperberg C, Kraft P, Krauter KS, Laakso M, Lind PA, Loukola A, Lutz SM, Madden PAF, Martin NG, McGue M, McQueen MB, Medland SE, Metspalu A, Mohlke KL, Nielsen JB, Okada Y, Peters U, Polderman TJC, Posthuma D, Reiner AP, Rice JP, Rimm E, Rose RJ, Runarsdottir V, Stallings MC, Stancakova A, Stefansson H, Thai KK, Tindle HA, Tyrfingsson T, Wall TL, Weir DR, Weisner C, Whitfield JB, Winsvold BS, Yin J, Zuccolo L, Bierut LJ, Hveem K, Lee JJ, Munafo MR, Saccone NL, Willer CJ, Cornelis MC, David SP, Hinds DA, Jorgenson E, Kaprio J, Stitzel JA, Stefansson K, Thorgeirsson TE, Abecasis G, Liu DJ, Vrieze S. Association studies of up to 1.2 million individuals yield new insights into the genetic etiology of tobacco and alcohol use. *Nat Genet* 2019;**51**:237-244.

21. Machiela MJ, Chanock SJ. LDlink: a web-based application for exploring population-specific haplotype structure and linking correlated alleles of possible functional variants. *Bioinformatics* 2015;**31**:3555-3557.

22. Wootton RE, Richmond RC, Stuijfzand BG, Lawn RB, Sallis HM, Taylor GMJ, Jones HJ, Zammit S, Davey Smith G, Munafo MR. Evidence for causal effects of lifetime smoking on risk for depression and schizophrenia: a Mendelian randomisation study. *Psychological Medicine* 2019:DOI: <https://doi.org/10.1017/S0033291719002678>.

23. Sudlow C, Gallacher J, Allen N, Beral V, Burton P, Danesh J, Downey P, Elliott P, Green J, Landray M, Liu B, Matthews P, Ong G, Pell J, Silman A, Young A, Sprosen T, Peakman T, Collins R. UK biobank: an open access resource for identifying the causes of a wide range of complex diseases of middle and old age. *PLoS Med* 2015;**12**:e1001779.

24. Malik R, Chauhan G, Traylor M, Sargurupremraj M, Okada Y, Mishra A, Rutten-Jacobs L, Giese AK, van der Laan SW, Gretarsdottir S, Anderson CD, Chong M, Adams HHH, Ago T, Almgren P, Amouyel P, Ay H, Bartz TM, Benavente OR, Bevan S, Boncoraglio GB, Brown RD, Jr., Butterworth AS, Carrera C, Carty CL, Chasman DI, Chen WM, Cole JW, Correa A, Cotlarciuc I, Cruchaga C, Danesh J, de Bakker PIW, DeStefano AL, den Hoed M, Duan Q, Engelter ST, Falcone GJ, Gottesman RF, Grewal RP, Gudnason V, Gustafsson S, Haessler J, Harris TB, Hassan A, Havulinna AS, Heckbert SR, Holliday EG, Howard G, Hsu FC, Hyacinth HI, Ikram MA, Ingelsson E, Irvin MR, Jian X, Jimenez-Conde J, Johnson JA, Jukema JW, Kanai M, Keene KL, Kissela BM, Kleindorfer DO, Kooperberg C, Kubo M, Lange LA, Langefeld CD, Langenberg C, Launer LJ, Lee JM, Lemmens R, Leys D, Lewis CM, Lin WY, Lindgren AG, Lorentzen E, Magnusson PK, Maguire J, Manichaikul A, McArdle PF, Meschia JF, Mitchell BD, Mosley TH, Nalls MA, Ninomiya T, O'Donnell MJ, Psaty BM, Pulit SL, Rannikmae K, Reiner AP, Rexrode KM, Rice K, Rich SS, Ridker PM, Rost NS, Rothwell PM, Rotter JI, Rundek T, Sacco RL, Sakaue S, Sale MM, Salomaa V, Sapkota BR, Schmidt R, Schmidt CO, Schminke U, Sharma P, Slowik A, Sudlow CLM, Tanislav C, Tatlisumak T, Taylor KD, Thijs VNS, Thorleifsson G, Thorsteinsdottir U, Tiedt S, Trompet S, Tzourio C, van Duijn CM, Walters M, Wareham NJ, Wassertheil-Smoller S, Wilson JG, Wiggins KL, Yang Q, Yusuf S, Bis JC, Pastinen T, Ruusalepp A, Schadt EE, Koplev S, Bjorkegren JLM, Codoni V, Civelek M, Smith NL, Tregouet DA, Christophersen IE, Roselli C, Lubitz SA, Ellinor PT, Tai ES, Kooner JS, Kato N, He J, van der Harst P, Elliott P, Chambers JC, Takeuchi F, Johnson AD, Sanghera DK, Melander O, Jern C, Strbian D, Fernandez-Cadenas I, Longstreth WT, Jr., Rolfs A, Hata J, Woo D, Rosand J, Pare G, Hopewell JC, Saleheen D, Stefansson K, Worrall BB, Kittner SJ, Seshadri S, Fornage M, Markus HS, Howson JMM, Kamatani Y, Debette S, Dichgans M, Malik R, Chauhan G, Traylor M, Sargurupremraj M, Okada Y, Mishra A, Rutten-Jacobs L, Giese AK, van der Laan SW, Gretarsdottir S, Anderson CD, Chong M, Adams HHH, Ago T, Almgren P, Amouyel P, Ay H, Bartz TM, Benavente OR, Bevan S, Boncoraglio GB, Brown RD, Jr., Butterworth AS, Carrera C, Carty CL, Chasman DI, Chen WM, Cole JW, Correa A, Cotlarciuc I, Cruchaga C, Danesh J, de Bakker PIW, DeStefano AL, Hoed MD, Duan Q, Engelter ST, Falcone GJ, Gottesman RF, Grewal RP, Gudnason V, Gustafsson S, Haessler J, Harris TB, Hassan A, Havulinna AS, Heckbert SR, Holliday EG, Howard G, Hsu FC, Hyacinth HI, Ikram MA, Ingelsson E, Irvin MR, Jian X, Jimenez-Conde J, Johnson JA, Jukema JW, Kanai M, Keene KL, Kissela BM, Kleindorfer DO, Kooperberg C, Kubo M, Lange LA, Langefeld CD, Langenberg C, Launer LJ, Lee JM, Lemmens R, Leys D, Lewis CM, Lin WY, Lindgren AG, Lorentzen E, Magnusson PK, Maguire J, Manichaikul A, McArdle PF, Meschia JF, Mitchell BD, Mosley TH, Nalls MA, Ninomiya T, O'Donnell MJ, Psaty BM, Pulit SL, Rannikmae K, Reiner AP, Rexrode KM, Rice K, Rich SS, Ridker PM, Rost NS, Rothwell PM, Rotter JI, Rundek T, Sacco RL, Sakaue S, Sale MM, Salomaa V, Sapkota BR, Schmidt R, Schmidt CO, Schminke U, Sharma P, Slowik A, Sudlow CLM, Tanislav C, Tatlisumak T, Taylor KD, Thijs VNS, Thorleifsson G, Thorsteinsdottir U, Tiedt S, Trompet S, Tzourio C, van Duijn CM, Walters M, Wareham NJ, Wassertheil-Smoller S, Wilson JG, Wiggins KL, Yang Q, Yusuf S, Amin N, Aparicio HS, Arnett DK, Attia J, Beiser AS, Berr C, Buring JE, Bustamante M, Caso V, Cheng YC, Choi SH, Chowhan A, Cullell N, Dartigues JF, Delavaran H, Delgado P, Dorr M, Engstrom G, Ford I, Gurpreet WS, Hamsten A, Heitsch L, Hozawa A, Ibanez L, Ilinca A, Ingelsson M, Iwasaki M, Jackson RD, Jood K, Jousilahti P, Kaffashian S, Kalra L, Kamouchi M, Kitazono T, Kjartansson O, Kloss M, Koudstaal PJ, Krupinski J, Labovitz DL, Laurie CC, Levi CR, Li L, Lind L, Lindgren CM, Lioutas V, Liu YM, Lopez OL, Makoto H, Martinez-Majander N, Matsuda K, Minegishi N, Montaner J, Morris AP, Muino E, Muller-Nurasyid M, Norrving B, Ogishima S, Parati EA, Peddareddygari LR, Pedersen NL, Pera J, Perola M, Pezzini A, Pileggi S, Rabionet R, Riba-Llena I, Ribases M, Romero JR, Roquer J, Rudd AG, Sarin AP, Sarju R, Sarnowski C, Sasaki M, Satizabal CL, Satoh M, Sattar N, Sawada N, Sibolt G, Sigurdsson A, Smith A, Sobue K, Soriano-Tarraga C, Stanne T, Stine OC, Stott DJ, Strauch K, Takai T, Tanaka H, Tanno K, Teumer A, Tomppo L, Torres-Aguila NP, Touze E, Tsugane S, Uitterlinden AG, Valdimarsson EM, van der Lee SJ, Volzke H, Wakai K, Weir D, Williams SR, Wolfe CDA, Wong Q, Xu H, Yamaji T, Sanghera DK, Melander O, Jern C, Strbian D, Fernandez-Cadenas I, Longstreth WT, Jr., Rolfs A, Hata J, Woo D, Rosand J, Pare G, Hopewell JC, Saleheen D, Stefansson K, Worrall BB, Kittner SJ, Seshadri S, Fornage M, Markus HS, Howson JMM, Kamatani Y, Debette S, Dichgans M. Multiancestry genome-wide association study of 520,000 subjects identifies 32 loci associated with stroke and stroke subtypes. *Nat Genet* 2018 **50**:524-537.

25. Larsson SC, Burgess S, Michaëlsson K. Smoking and stroke: a Mendelian randomization study. *Ann Neurol* 2019;**86**:468-471.

26. Higgins JP, Thompson SG. Quantifying heterogeneity in a meta-analysis. *Stat Med* 2002;**21**:1539-1558.

27. Burgess S, Thompson SG. Multivariable Mendelian randomization: the use of pleiotropic genetic variants to estimate causal effects. *Am J Epidemiol* 2015;**181**:251-260.

28. Ntalla I, Kanoni S, Zeng L, Giannakopoulou O, Danesh J, Watkins H, Samani NJ, Deloukas P, Schunkert H, Group UKBCCCW. Genetic Risk Score for Coronary Disease Identifies Predispositions to Cardiovascular and Noncardiovascular Diseases. *J Am Coll Cardiol* 2019;**73**:2932-2942.

29. Burgess S, Bowden J, Fall T, Ingelsson E, Thompson SG. Sensitivity analyses for robust causal inference from Mendelian randomization analyses with multiple genetic variants. *Epidemiology* 2017;**28**:30-42.

30. Spiller W, Davies NM, Palmer TM. Software application profile: mrrobust—a tool for performing two-sample summary Mendelian randomization analyses *Int J Epidemiol* 2019;**48**:684–690.

31. Yavorska OO, Burgess S. MendelianRandomization: an R package for performing Mendelian randomization analyses using summarized data. *Int J Epidemiol* 2017;**46**:1734-1739.

32. Larsson SC, Bäck M, Rees JMB, Mason AM, Burgess S. Body mass index and body composition in relation to 14 cardiovascular conditions in UK Biobank: a Mendelian randomization study. *Eur Heart J pii: ehz388 doi: 101093/eurheartj/ehz388 [Epub ahead of print]* 2019.

33. Rigotti NA, Clair C. Managing tobacco use: the neglected cardiovascular disease risk factor. *Eur Heart J* 2013;**34**:3259-3267.

34. Kamio Y, Miyamoto T, Kimura T, Mitsui K, Furukawa H, Zhang D, Yokosuka K, Korai M, Kudo D, Lukas RJ, Lawton MT, Hashimoto T. Roles of Nicotine in the Development of Intracranial Aneurysm Rupture. *Stroke* 2018;**49**:2445-2452.

35. Baron JA, La Vecchia C, Levi F. The antiestrogenic effect of cigarette smoking in women. *Am J Obstet Gynecol* 1990;**162**:502-514.

36. Michnovicz JJ, Hershcopf RJ, Haley NJ, Bradlow HL, Fishman J. Cigarette smoking alters hepatic estrogen metabolism in men: implications for atherosclerosis. *Metabolism* 1989;**38**:537-541.

37. Michnovicz JJ, Hershcopf RJ, Naganuma H, Bradlow HL, Fishman J. Increased 2-hydroxylation of estradiol as a possible mechanism for the anti-estrogenic effect of cigarette smoking. *N Engl J Med* 1986;**315**:1305-1309.

38. Connor Gorber S, Schofield-Hurwitz S, Hardt J, Levasseur G, Tremblay M. The accuracy of self-reported smoking: a systematic review of the relationship between self-reported and cotinine-assessed smoking status. *Nicotine Tob Res* 2009;**11**:12-24.

39. Burgess S, Davies NM, Thompson SG. Bias due to participant overlap in two-sample Mendelian randomization. *Genet Epidemiol* 2016;**40**:597-608.

40. Piepoli MF, Hoes AW, Agewall S, Albus C, Brotons C, Catapano AL, Cooney MT, Corra U, Cosyns B, Deaton C, Graham I, Hall MS, Hobbs FDR, Lochen ML, Lollgen H, Marques-Vidal P, Perk J, Prescott E, Redon J, Richter DJ, Sattar N, Smulders Y, Tiberi M, van der Worp HB, van Dis I, Verschuren WMM, Binno S, Group ESCSD. 2016 European Guidelines on cardiovascular disease prevention in clinical practice: The Sixth Joint Task Force of the European Society of Cardiology and Other Societies on Cardiovascular Disease Prevention in Clinical Practice (constituted by representatives of 10 societies and by invited experts)Developed with the special contribution of the European Association for Cardiovascular Prevention & Rehabilitation (EACPR). *Eur Heart J* 2016;**37**:2315-2381.
